# Identification of Nontuberculous Mycobacteria in Drinking Water in Cali, Colombia

**DOI:** 10.3390/ijerph18168451

**Published:** 2021-08-10

**Authors:** Andrés F. Dávalos, Pamela K. Garcia, Carolina Montoya-Pachongo, Andrea Rengifo, Daniela Guerrero, Lorena Díaz-Ordoñez, Gustavo Díaz, Beatriz E. Ferro

**Affiliations:** 1 Facultad de Ciencias Naturales, Universidad Icesi, Calle 18 # 122-135, Cali 760031, Valle del Cauca, Colombia; afdavalos@icesi.edu.co (A.F.D.); andrearbernate@hotmail.com (A.R.); laura3027@hotmail.com (D.G.); diaz.gustavo2011@gmail.com (G.D.); 2 Department of Pathology and Laboratory Medicine, School of Medicine, Emory University, 201 Dowman Dr, Atlanta, GA 30322, USA; pam2814@gmail.com; 3 Grupo de Investigación Microbiología, Industria y Medio Ambiente, Universidad Santiago de Cali, Calle 5 # 62-00, Cali 760035, Valle del Cauca, Colombia; 4 School of Civil Engineering, University of Leeds, Woodhouse Lane, Leeds LS2 9JT, UK; C.MontoyaPachongo@leeds.ac.uk; 5 Cognita Links, Cra. 65 No. 13B-125, Cali 760033, Valle del Cauca, Colombia; 6 Facultad de Ciencias de la Salud, Universidad Icesi, Calle 18 # 122-135, Cali 760031, Valle del Cauca, Colombia; lldiaz@icesi.edu.co; 7 Centro Internacional de Entrenamiento e Investigaciones Médicas, CIDEIM, Calle 18 # 122-135, Cali 760031, Valle del Cauca, Colombia

**Keywords:** antibiotic resistance, drinking water, nontuberculous mycobacteria, public health

## Abstract

Nontuberculous mycobacteria (NTM) are ubiquitous microorganisms naturally resistant to antibiotics and disinfectants that can colonize drinking water supply systems. Information regarding the spread of NTM in specifically South America and Colombia is limited. We aimed to identify and characterize NTM present in tap water samples from Cali, Colombia. Drinking water samples and faucet biofilm swabs were collected in 18 places, including the city’s three main water treatment plants (WTPs). Filter-trapped material and eluates (0.45 μm) from swab washes were plated in 7H11 agar plates. Suspected colonies were evaluated microscopically, and NTM species were identified based on the *rpoB* gene. Antibiotic susceptibility testing was also performed. Fifty percent (9/18) of sampling points were positive for NTM (including two WTPs), from which 16 different isolates were identified: *Mycobacterium mucogenicum* (8/16), *M. phocaicum* (3/16), *M. chelonae* (2/16), *M. mageritense* (2/16), and *M. fortuitum* (1/16), all rapidly growing mycobacteria. A susceptibility profile was obtained from 68.75% (11/16) of the isolates. *M. chelonae* was the most resistant species. All NTM isolated are potentially responsible for human diseases; our findings might provide a baseline for exploring NTM transmission dynamics and clinical characterization, as well as potential associations between NTM species found in drinking water and isolates from patients.

## 1. Introduction

Nontuberculous mycobacteria (NTM) are aerobic, nonmotile, and ubiquitous organisms found in water and soil. Collectively, their cell wall composition, biofilm formation ability, resistance to high temperature, and low pH allow these bacteria to be naturally resistant to antibiotics and disinfectants, as well as colonize different moist environments, including drinking water supply systems [1,2,3]. NTM species have been isolated from both patients and environmental sources; more than 200 species of NTM have been characterized by molecular biology techniques [4,5]. The most common NTM associated with human infection include *Mycobacterium avium* complex (MAC), *M. kansasii*, *M. xenopi*, and *M. malmoense* from slowly growing NTM and *M. abscessus* complex, *M. chelonae*, and *M. fortuitum* from rapidly growing NTM. Nevertheless, their frequency and distribution vary, which makes the study and determination of the local distribution of NTM species in potential sources of infection essential.

The pathogenic potential of NTM has been recognized since the 1950s; however, only recently has the interest in NTM as etiological agents of opportunistic infectious diseases started increasing, not only in immunocompromised or elderly people, but also in the immunocompetent population [6,7,8,9,10]. In fact, 22 studies performed between 1946 and 2014 reported a 94% increase in infections caused by NTM [11]. Most of the information about NTM as agents that cause disease has been published in the United States and Europe [12,13], and the frequency of isolation and clinical relevance of NTM species vary among continents, countries, and even cities [14,15]. However, these publications coincide on two facts: (1) the prevalence and incidence of NTM infections has increased in the past decade [12,16,17], and (2) there is an imperative necessity of conducting ecological and epidemiological studies to reveal possible associations between the distribution of NTM species and environmental reservoirs, clinical presentations, and host susceptibility [17,18,19].

In Colombia, the prevalence and distribution of NTM have been scarcely studied. In 2010, Correa et al. published a study including 29 cases of NTM subcutaneous infections occurring after mesotherapy, most of which were associated with *M. chelonae*, followed by *M. abscessus* and *M. fortuitum*, from private dermatological practices in the city of Medellín [20]. Llerena et al. reported that infections caused by NTM were mainly associated with the MAC complex, followed by *M. abscessus* and *M. fortuitum.* According to the Laboratorio Nacional de Referencia in Colombia, between 2012 and 2016, the most common form of NTM-associated disease was pulmonary [21]. In addition, an exploratory study carried out in the city of Cali also showed that 1.5% of sputum cultures from patients under study for active tuberculosis between 2014 and 2017 exhibited NTM growth, with *M. fortuitum* and *M. abscessus* being the most commonly identified species [22]. However, little is known about the primary source of those NTM in Colombia, and environmental sources typically colonized with NTM, such as local water supplies, could be playing an important role in human infection and disease.

In this study, we aimed to determine the presence of NTM in tap water samples from Cali, Colombia. We also determined the susceptibility profile of isolated bacteria. The knowledge generated might provide a baseline for future research in the field of NTM transmission dynamics and clinical characterization, as well as establish associations between NTM species found in drinking water or specific water sources and species isolated from patients. Thus, the detection of potential associations would inform strategies and policies directed toward preventing NTM infections in susceptible people.

## 2. Materials and Methods

Drinking water samples were collected from buildings in the city of Cali, Colombia. This city is located 995 m above sea level, and the annual average temperature is 24.5 °C (23.8–25.1 °C). The water supply system is composed of four subsystems that originate from four surface water sources and five treatment facilities. The Pance River subsystem supplies drinking water to 0.4% of the total customers, the Meléndez River subsystem supplies to 3.8%, the Cali River subsystem supplies to 10.8%, and the Cauca River subsystem supplies to 85%. Water treatment plants (WTPs) apply conventional processes in treating raw water (coagulation, flocculation, sedimentation, filtration, and final disinfection with chlorine). The two biggest WTPs, Puerto Mallarino and Río Cauca (both compose the Cauca River subsystem), also use chlorine for primary disinfection. The distribution system operates by gravity, pumping, or a combination of both, and includes 2951 km of pipelines, 10 service reservoirs, 28 storage tanks, and 19 pumping stations to deliver water to about 657,000 customers. In 2017, the water utility began partitioning the distribution network; the goal was to materialize 85 hydraulic sectors. Currently, the project is 62% completed (53 hydraulics sectors in operation as of 2021).

**Figure 1:**
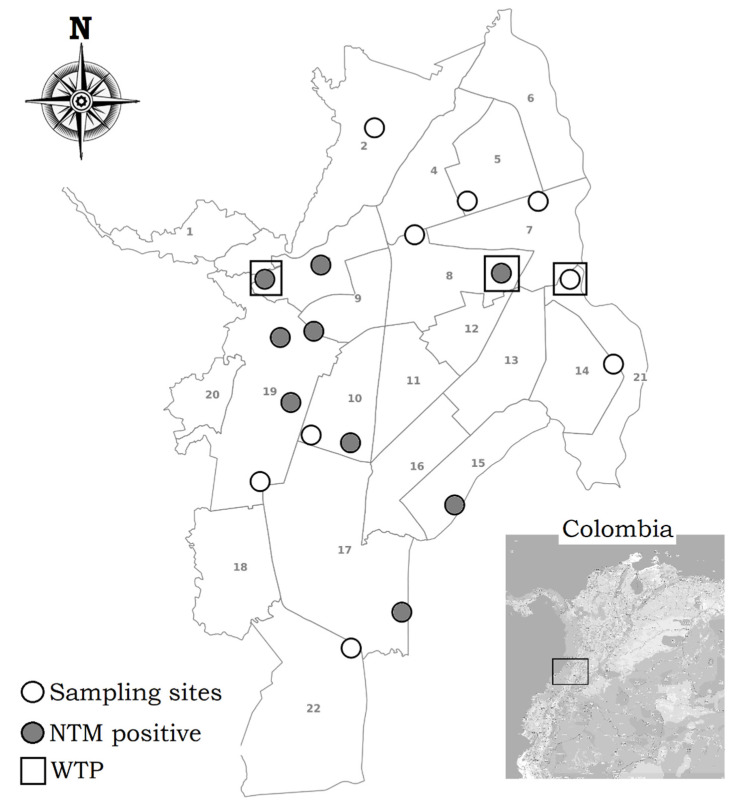
Location of sampling points in the city of Cali, Colombia. WTP, water treatment plant; NTM, nontuberculous mycobacteria. Numbers 1 to 22 represent division of the city in communes.

Table 1 shows that 18 points were sampled in total; three samples were collected from the WTP outlets, and the remaining samples were obtained from buildings located in different neighborhoods covering the four corners of the city; just one sample at each point and time was collected (Figure 1). Thirteen premises were used for housing, and the other two were commercial establishments; several of them had domestic storage tanks. Before performing any manipulation of the faucet, we sampled biofilms by introducing a polystyrene PurFlock^®^ ULTRA (Puritan, Guilford, ME, USA) sterile swab in the spout of the faucet and making circular movements against the walls for approximately 30 s. To avoid collection of stagnant water, the faucets were allowed to run for 15 s before the sample was collected; 2 L of water were collected in sterile containers. Immediately after collection, both swabs and water samples were transported to the microbiology laboratory of Universidad Icesi and processed within the following 2 h. Sampling took place at two different moments for each sampling point between the months of June and October 2018; no replicates were processed. During this period, the monthly precipitation, according to four meteorological stations, ranged from 14 mm to 133 mm, with an average of 54 ± 33 mm.

Samples were processed following the protocol described by Tichenor et al. and Falkinham et al. [23,24]. The swabs were plated on two Middlebrook 7H11 agar plates and incubated at 30 °C and 37 °C. The temperature, pH, and conductivity were measured in all water samples, followed by filtration through a sterile membrane with a pore size of 0.45 µm. Both swabs and membranes were then transferred to a 50-mL tube containing cetylpyridinium chloride (0.005%) for 30-min decontamination. The swabs were removed using a sterile tweezer, and the filter membranes were scraped with sterile blades to detach any bacterial cells from the surface. Afterward, the filter membrane was removed from the 50-mL tube using a sterile tweezer and split in half using sterile scissors. Both membrane fragments were placed on a Middlebrook 7H11 agar separately for incubation at 30 °C and 37 °C. The tubes with cetylpyridinium chloride containing the swabs and membrane scraping residues were spun down at 3.5k RCF for 20 min, the supernatant was removed, and the pellet was washed once in 200 µL of sodium chloride (0.85%). After washing, the pellet was spun down at 3.5k RCF for 10 min, and 100 µL was plated on Middlebrook 7H11 agar. Each plate was incubated at 30 °C and 37 °C as previously mentioned; the Middlebrook 7H11 agar plates were monitored for 20 days.

Suspected colonies, which were mostly dry, had uneven borders, and varied in color from cream to yellow or orange, were prepared for microscopy examination using Gram and Ziehl–Neelsen staining. We extracted the genomic DNA of all acid-fast colonies using PrepMan Ultra Sample Preparation Reagent (Applied Biosystems, Waltham, MA, USA), according to the manufacturer’s recommendations.

To identify NTM species, a set of primers was used to amplify the beta subunit of RNA polymerase *rpoB* gene (723 bp): MycoF: 5′-GGCAAGGTCACCCCGAAGGG-3′ and MycoR: 5′-AGCGGCTGCTGGGTGATCATC-3′, previously reported by Adékambi et al. [25]. The polymerase chain reaction conditions included an initial denaturation for 1 min at 95 °C, followed by 35 cycles of: denaturation for 30 s at 94 °C, primer annealing for 30 s at 64 °C, and extension for 90 s at 72 °C. The final extension was held for 5 min at 72 °C. Sanger sequencing was performed using the 3500 Genetic Analyzer and the BigDye™ Terminator v3.1 Cycle Sequencing Kit (Thermo Fisher Scientific, Waltham, MA, USA), according to the fabricant’s recommendations. Data analysis was performed using the Sequencing Analysis v 6.0 software (Thermo Fisher Scientific Waltham, MA, USA) and the Basic Local Alignment Search Tool. To construct a phylogenetic tree, Mega software (version 7) was used. The methodology used was the maximum likelihood based on the Tamura–Nei model, and the stability of the phylogram was evaluated by parsimony bootstrapping with 500 simulations.

For antibiotic susceptibility testing, we used commercial broth microdilution panels (RAPMYCO Sensititre™, Trek Diagnostics/Thermo Fisher, Waltham, MA, USA) following the manufacturer’s recommendations. This panel tests the activities of amikacin, cefoxitin, ciprofloxacin, clarithromycin, doxycycline, imipenem, linezolid, sulfamethoxazole, moxifloxacin, and tobramycin, as recommended by the CLSI [26].

## 3. Results

A total of 16 NTM isolates were identified in water samples from nine of the 18 points evaluated (Figure 1). These positive locations corresponded to two WTPs (11.1%), which supply drinking water for 25% of Cali’s inhabitants: four (22.2%) domestic premises, one (5.6%) commercial premise, and two (11.1%) public buildings. All water samples met the criteria established by Colombian regulations for the physicochemical properties for pH and conductivity (Table 2). None of the biofilm swabs were positive for NTM isolation.

**Figure 2:**
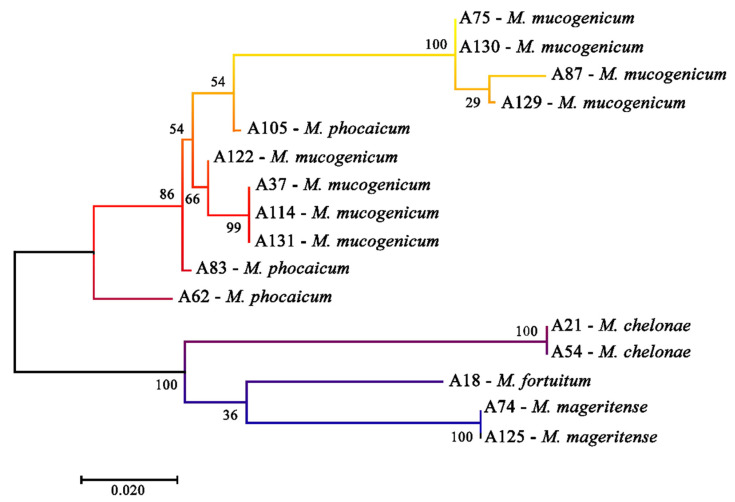
Molecular phylogenetic analysis by maximum likelihood method.

The 16 NTM isolates were identified as five different species: *M. mucogenicum*, *M. phocaicum*, *M. fortuitum*, *M. chelonae*, and *M. mageritense*, all rapidly growing mycobacteria. The most frequently identified species was *M. mucogenicum*, which was detected in eight isolates (50%), followed by *M. phocaicum* in three (18.75%)*, M. mageritense* in two (12.5%), *M. chelonae* in two (12.5%), and *M. fortuitum* in one isolate (6.25%). Figure 2 represents the phylogenetic analysis based on the *rpoB* sequence to determine the species. The percentage of trees in which the associated taxa clustered together is shown next to the branches. The tree is drawn to scale, with the lengths of the branches measured in the number of substitutions per site. The analysis involved 16 nucleotide sequences. All positions containing gaps and missing data were eliminated. The final dataset consisted of 592 positions. Evolutionary analyses were conducted in MEGA 7.0.

There was variation in the species distribution by the type of sampling point; *M. mucogenicum* strains were obtained with a relative frequency of 12.5% from WTPs, 18.5% from domestic premises, 12.5% from a public buildings, and 6.5% from commercial premises. Three *M. phocaicum* isolates were obtained with a relative frequency of 12.5% from domestic and 6.25% from commercial premises; two *M. chelonae* isolates that corresponded to a relative frequency of 12.5% were obtained from domestic premises; two *M. mageritense* isolates were obtained from a WTP (6.5%) and a domestic premise (6.5%); and one *M. fortuitum* isolate was obtained from a WTP (6.5%).

Six of the 13 samples collected from the Cauca River subsystem and all (three) from the Cali River subsystem were positive for the presence of NTM. The data suggested that NTM obtained from the Cauca River subsystem were more diverse than those identified in the Cali River subsystem (five species vs. two species, respectively). The species *M. mucogenicum*, *M. chelonae*, *M. phocaicum*, *M. mageritense*, and *M. fortuitum* were identified from the Cauca River subsystem samples, and *M. mucogenicum* and *M. phocaicum* were identified from the Cali River subsystem samples.

Although the use of susceptibility testing is generally recommended only for clinically significant mycobacteria, we performed a susceptibility exploration for 11 of 16 isolates (68.75%), which revealed that, in general, *M. chelonae* and *M. mageritense* were the species with the most resistant profile to the antibiotics tested, and that doxycycline, ciprofloxacin, and tobramycin were the antibiotics for which more resistant isolates were detected (Table 3). The remaining five isolates could not be evaluated because of budget constraints.

## 4. Discussion

This exploratory study is the first to report the isolation, characterization, and antibiotic susceptibility profile of NTM from drinking water samples in Cali, Colombia. Fifty percent (9/18) of the sampling points in our study were positive for NTM, from which we identified 16 different isolates. Several studies have identified drinking water as one of the most common vehicles and distribution systems, as well as reservoirs, for NTM. Pérez and Martínez in Ciudad de México managed to isolate NTM species in 16% of the drinking water samples analyzed [28]; Tortone and collaborators in Ciudad de la Pampa isolated NTM in 37.5% of the samples analyzed [29]; and Oriani and colleagues in Bahia Blanca, Argentina, isolated NTM in 51.6% (64/124) of the drinking water samples analyzed [30]. Donohue et al. [31] collected 272 samples from 40 geographically dispersed sites in the United States and detected culturable mycobacteria at least once in 98% of faucets. Collectively, all of these studies demonstrated that drinking water and its distribution networks are common habitats for NTM [18,32]. Although there are few reports describing the distribution of NTM in South America, Zweijpfenning et al. reported that the species most frequently found in this part of the world are those of the MAC complex, with a frequency of 34%, followed by *M. kansasii* with 17%, *M. gordonae* with 15%, and *M. fortuitum* with 9% [14]. However, our findings coincide with the results of the study by Pérez-Martínez [28], who reported that *M. mucogenicum* was the most frequently isolated species, followed by *M. fortuitum*, although with less frequency. Studies carried out in Germany, Australia, and France reported higher frequencies of isolation of NTM from drinking water than those reported in South America, with percentages ranging between 57% and 72%, respectively [33,34,35]. The difference between the two continents could be associated with the underreporting, technical, and resource limitations in South America, which might have led to an analysis of a smaller number of samples [34]. These results could also be explained by the age and materials of the distribution networks of European and Australian cities, considering that their cities are older, and therefore, their pipes could be older, which generates particular conditions that lead to certain species of bacteria proliferating more than others [36,37,38,39].

All isolated species found in this study corresponded to rapidly growing mycobacteria, which, according to Hoefsloot et al., have been found with greater frequency in the United Kingdom, Greece, and Canada [40]. Overall, the NTM phylogenetic tree shows three distinct groups. The first group included four closely related isolates of *M. mucogenicum*, although they were obtained from different WTPs. The second group included isolates of *M. mucogenicum* that were not closely related to group 1, but these were closely related to other isolates of *M. phocaicum*. These findings suggest that strains from different WTPs have undergone gene rearrangements. Finally, the tree also showed a third group that included one isolate of *M. fortuitum* and two identical isolates of *M. mageritense* and *M chelonae*, which suggests an isolation of the same strains.

The NTM species isolated in this study are potentially responsible for human diseases. *M. fortuitum* and *M. chelonae* have been associated mainly with skin and soft tissue infections [41,42,43]; however, there have been cases reported of pulmonary disease caused by these organisms [44,45]. *M. mucogenicum* has been associated with catheter-related sepsis [46], pulmonary infections [47], and infections at the central nervous system level [48]. In addition, drug-resistant isolates of *M. mucogenicum* have been reported from hospital surfaces [49]. *M. phocaicum* has been associated with lung disease acquired from hot tubs and pool water [50,51] and, at a lower rate, with bloodstream infection [52]. *M. mageritense* has been associated with pulmonary and skin infections. However, the mere presence of NTM is not directly associated with human disease, and its pathogenic potential remains to be elucidated in our setting.

*M. fortuitum* was the most susceptible isolate, showing resistance only to tobramycin and clarithromycin. Intrinsic resistance to clarithromycin conferred by the inducible ermmethylase gene *erm* (39) is common in *M. fortuitum* [53,54]. However, additional molecular studies are required to provide a deeper characterization of macrolide resistance in the isolated strain. On the other hand, *M. chelonae* has been associated with high levels of drug resistance compared with to other NTM. In fact, one strain isolated in this study showed resistance to six of the 10 drugs tested. Scarce information on drug susceptibility for the remaining species prevents us from discussing the susceptibility findings, which in any case are mostly important for clinical practice, and should be considered for clinical importance studies.

Although all species were isolated from drinking water samples, 75% (12/16) were isolated from faucets located in houses, apartments, or commercial premises; the remaining 25% (4/16), corresponding to *M. mucogenicum* (2), *M. fortuitum* (1), and *M. mageritense* (1), were isolated from two WTPs that supply drinking water to 25% of customers. Such plants take raw water from two different surface water sources: the Cauca River and Cali River. The Cauca River is characterized by an advanced degree of environmental deterioration, as there are various uses of soil and water in its basin, such as licit and illicit crops, urban settlements, presence of manufacturing industries, livestock, fishing, extraction of sand, and recreational use. All of the above contribute to pesticides, micropollutants, heavy metals, organic and inorganic matter, low dissolved oxygen, and pathogenic microorganisms, among others [55]. On the contrary, the Cali River is a smaller source of water with better conservation conditions, since it originates in the protected area of Farallones National Park. Moreover, the influence of urban settlements is more limited, and there is little to no large-scale industrial activity in its middle basin, where the catchment of the Río Cali WTP is located. However, in 2015, authorities detected and intervened in illegal mining activities for gold extraction, and in its lower basin, the quality of water shows a notorious deterioration due to the discharge of domestic wastewater from the urban and rural areas of the city of Cali [56]. We do not know how the abovementioned dynamic in rivers may affect NTM species distribution in the city.

The detection of NTM in WTPs after water treatment would partially explain the presence of NTM in other sampling points. Just three of the five species characterized were isolated from WTPs. Several factors could have an influence on the detection of potential pathogens in treated water before or after passing through the distribution systems, such as resistance to purification agents and further bacterial regrowth in distribution systems, formation of biofilm in distribution systems after surviving purification agents, contamination in the domestic pumping sites and storage tanks, and cross-contamination of the distribution networks or in the plumbing systems during maintenance activities, which cause a loss of physical integrity of the supply system [57,58].

Genotypic and structural characteristics of NTM make them resistant to purification treatments and enable them to proliferate, travel, and spread through drinking water distribution networks, including plumbing systems. In addition, their detection, identification, and quantification are not microbiological parameters included for the control and surveillance of the quality of drinking water worldwide. Therefore, entities that are in charge of public health policies should be sensitized to the importance of investigating the real risks that this group of bacteria present for human health, mainly in plumbing systems, where susceptible people live, either due to underlying diseases or some type of immunosuppression. Preventive measures should be implemented to help mitigate the spread of NTM in high-risk populations, including the implementation of commercial water purification systems based on filtration and ultraviolet light, particularly for water intended for ingestion, food preparation, and personal hygiene, as well as advice to avoid saunas, swimming pools, and hot tubs, which are known sources of NTM.

## 5. Limitations

The absence of slow-growing NTM isolates may be attributed to conditions favored by the isolation and culture techniques used, which could have generated an environment that was not conducive to the development of slow-growing NTM, rather than a real absence of them. Among the conditions that we think could have interfered with the development of slow-growing NTM is the overgrowth of fast-growing NTM and other groups of bacteria and fungi, as well as the dehydration of the culture media after an incubation period of two to three weeks. These observations should be considered for future studies. Similarly, despite the common presence of NTM on biofilms, we did not obtain any isolates from the swabs used to sample faucet biofilms, perhaps as a result of the methodological constraints. Because of the limitations of the sample size, we were unable to determine the relationship of storage tanks to the presence of NTM. Finally, since most of the samples were collected during a period usually considered summer, it was not possible to establish the potential impact of climate on the results obtained.

## 6. Conclusions

To our knowledge, few studies have been reported on the presence of NTM in drinking water in Latin America. This work represents a contribution to improve the knowledge regarding the presence of NTM in drinking water in the city of Cali, Colombia. The species of NTM identified in the drinking water collected from plumbing systems were *M. mucogenicum*, *M. mageritense, M. phocaicum*, and *M. chelonae,* and those collected from the outlet of two WTPs were *M. mucogenicum*, *M. mageritense*, and *M. fortuitum*. Samples collected from the Cauca River subsystem, which is supplied by a very contaminated river, presented more NTM in comparison with samples from the Cali River subsystem: five species of NTM were isolated from the former, whereas two species was isolated from the latter.

More research must be conducted to determine the presence, abundance, and infection capability of NTM in plumbing systems from buildings that host vulnerable populations, such as elderly individuals, children, and immunocompromised people. In addition, education and further, more advanced treatment processes should be implemented in such facilities to inactivate NTM and other opportunistic water-borne pathogens.

## Figures and Tables

**Table 1:** Sampling points in the study area.

Sampling Point ID	WTP/Neighborhood	Supplied by WTP	Storage Tank	Floor in the Building	NTM Species Isolated
1	WTP Río Cali	—	—	—	A87-*M. mucogenicum*
2	WTP Río Cauca	—	—	—	A18-*M. fortuitum* A75-*M. mucogenicum* A74-*M. mageritense*
3	WTP Puerto Mallarino	—	—	—	—
4	Urbanización Río Lili	Puerto Mallarino * Río Cauca	Yes	4	—
5	Los Cámbulos	Río Cauca * Puerto Mallarino	Yes	2	A21-*M. chelonae* A54-*M. chelonae*
6	Industrial	Río Cauca * Puerto Mallarino	No	1	
7	Las Granjas	Puerto Mallarino	No	1	A62-*M. phocaicum*
8	Panamericano	Puerto Mallarino * Río Cauca	No	1	—
9	Refugio	Río Cauca * Puerto Mallarino	Yes	6	—
10	Salomia	Puerto Mallarino	No	2	—
11	San Fernando	Río Cali	No	1	A37-*M. mucogenicum* A83-*M. phocaicum*
12	Vipasa **	Puerto Mallarino Río Cauca	No	1	—
13	Alameda	Río Cauca * Puerto Mallarino	No	1	A105-*M. phocaicum*
14	Valle del Lili	Puerto Mallarino	Yes	3	A122-*M. mucogenicum*
15	Alfonso López **	Puerto Mallarino Río Cauca	No	2	—
16	San Pedro	Río Cali	Yes	1	A114-*M. mucogenicum* A130-*M. mucogenicum* A131-*M. mucogenicum*
17	Puertas del Sol	Puerto Mallarino	No	1	—
18	Ciudad Córdoba	Puerto Mallarino	No	1	A125-*M. mageritense* A129-*M. mucogenicum*

WTP, water treatment plant; NTM, nontuberculous mycobacteria. * Predominant plant supplying the sampling point. ** Some premises may receive water from both WTPs.

**Table 2:** Physicochemical properties of drinking water samples.

Parameter	Median (Interquartile Range)	Mean (Standard Deviation)	Accepted Parameters in Colombian Regulations [27]
Temperature (°C) (*n* = 33)	25.2 (24.6–25.4)	24.9 (1.1)	Not regulated
pH (*n* = 33)	7.3 (7.0–7.9)	7.4 (0.5)	6.5–9.0
Conductivity (uS/cm) (*n* = 32)	136.4 (128.3–150.4)	139.8 (18.8)	Up to 1000

**Table 3:** Minimum inhibitory concentration for nontuberculous mycobacteria isolated from drinking water in Cali, Colombia.

Antimicrobial Agent	MIC (mg/L) Breakpoints			MIC (mg/L) for RGM Isolates				
	S	I	R	*M. fortuitum* A18	*M. chelonae* A21, A54	*M. mucogenicum* A37,A75, A87, A114	*M. phocaicum* A62, A83, A105	*M. mageritense* A74
Amikacin	≤16	32	≥64	S	I, S	S, S, S, S	S, S, S	S
Tobramycin	≤4	8	≥16	R	S, S	I, R, R, R	R, S, I	R
Doxycycline	≤1	2–8	≥16	S	R, R	R, R, S, S	I, R, S	R
Ciprofloxacin	≤1	2	≥4	S	R, I	S, R, S, S	R, R, R	R
Moxifloxacin	≤1	2	≥4	S	R, R	S, I, S, S	S, S, S	S
Clarithromycin	≤2	4	≥8	R	S, S	S, S, S, R	S, S, S	R
Linezolid	≤8	16	≥32	S	R, S	S, S, S, R	S, S, S	S
Imipenem	≤4	8	≥16	S	R, R	S, S, I, S	I, S, S	R
Cefoxitin	≤16	32–64	≥128	I	R, R	S, S, S, S	I, S, S	I
Sulfamethoxazole	≤32	-	≥64	S	R, R	S, S, S, R	S, S, S	-

MIC, minimum inhibitory concentration; S, susceptible; I, intermediate; R, resistant; RGM, rapidly growing mycobacteria.

## Data Availability

Data to be published.

## References

[1] Jarlier V., Nikaido H. (1994). Mycobacterial cell wall: Structure and role in natural resistance to antibiotics. FEMS Microbiol. Lett..

[2] Faria S., Joao I., Jordao L. (2015). General Overview on Nontuberculous Mycobacteria, Biofilms, and Human Infection. J. Pathog..

[3] Gebert M.J., Delgado-Baquerizo M., Oliverio A.M., Webster T.M., Nichols L.M., Honda J.R., Chan E.D., Adjemian J., Dunn R.R., Fierer N. (2018). Ecological Analyses of Mycobacteria in Showerhead Biofilms and Their Relevance to Human Health. MBio.

[4] Parte A.C. (2018). LPSN—List of Prokaryotic names with Standing in Nomenclature (bacterio.net), 20 years on. Int. J. Syst. Evol. Microbiol..

[5] Tortoli E. (2003). Impact of genotypic studies on mycobacterial taxonomy: The new mycobacteria of the 1990s. Clin. Microbiol. Rev..

[6] Griffith D.E., Aksamit T., Brown-Elliott B.A., Catanzaro A., Daley C., Gordin F., Holland S.M., Horsburgh R., Huitt G., Iademarco M.F. (2007). An official ATS/IDSA statement: Diagnosis, treatment, and prevention of nontuberculous mycobacterial diseases. Am. J. Respir. Crit. Care Med..

[7] To K., Cao R., Yegiazaryan A., Owens J., Venketaraman V. (2020). General Overview of Nontuberculous Mycobacteria Opportunistic Pathogens: Mycobacterium avium and Mycobacterium abscessus. J. Clin. Med..

[8] Prince D.S., Peterson D.D., Steiner R.M., Gottlieb J.E., Scott R., Israel H.L., Figueroa W.G., Fish J.E. (1989). Infection with Mycobacterium avium complex in patients without predisposing conditions. N. Engl. J. Med..

[9] Henry M.T., Inamdar L., O’Riordain D., Schweiger M., Watson J.P. (2004). Nontuberculous mycobacteria in non-HIV patients: Epidemiology, treatment and response. Eur. Respir. J..

[10] Donohue M.J. (2018). Increasing nontuberculous mycobacteria reporting rates and species diversity identified in clinical laboratory reports. BMC Infect. Dis..

[11] Brode S.K., Daley C.L., Marras T.K. (2014). The epidemiologic relationship between tuberculosis and non-tuberculous mycobacterial disease: A systematic review. Int. J. Tuberc. Lung Dis..

[12] van Ingen J., Bendien S.A., de Lange W.C., Hoefsloot W., Dekhuijzen P.N., Boeree M.J., van Soolingen D. (2009). Clinical relevance of non-tuberculous mycobacteria isolated in the Nijmegen-Arnhem region, The Netherlands. Thorax.

[13] Adjemian J., Olivier K.N., Seitz A.E., Holland S.M., Prevots D.R. (2012). Prevalence of nontuberculous mycobacterial lung disease in U.S. Medicare beneficiaries. Am. J. Respir. Crit. Care Med..

[14] Zweijpfenning S.M.H., Ingen J.V., Hoefsloot W. (2018). Geographic Distribution of Nontuberculous Mycobacteria Isolated from Clinical Specimens: A Systematic Review. Semin. Respir. Crit. Care Med..

[15] Schiff H.F., Jones S., Achaiah A., Pereira A., Stait G., Green B. (2019). Clinical relevance of non-tuberculous mycobacteria isolated from respiratory specimens: Seven year experience in a UK hospital. Sci. Rep..

[16] Prevots D.R., Loddenkemper R., Sotgiu G., Migliori G.B. (2017). Nontuberculous mycobacterial pulmonary disease: An increasing burden with substantial costs. Eur. Respir. J..

[17] Rivero-Lezcano O.M., González-Cortés C., Mirsaeidi M. (2019). The unexplained increase of nontuberculous mycobacteriosis. Int. J. Mycobacteriol..

[18] Honda J.R., Virdi R., Chan E.D. (2018). Global Environmental Nontuberculous Mycobacteria and Their Contemporaneous Man-Made and Natural Niches. Front. Microbiol..

[19] Chou M.P., Clements A.C., Thomson R.M. (2014). A spatial epidemiological analysis of nontuberculous mycobacterial infections in Queensland, Australia. BMC Infect. Dis..

[20] Correa N.E., Cataño J.C., Mejía G.I., Realpe T., Orozco B., Estrada S., Vélez A., Vélez L., Barón P., Guzmán A. (2010). Outbreak of mesotherapy-associated cutaneous infections caused by Mycobacterium chelonae in Colombia. Jpn. J. Infect. Dis..

[21] Llerena C., Valbuena A., Zabaleta A.P. (2018). Mycobacterioses identified in the National Reference Laboratory of Colombia from 2012 to 2016. Biomedica.

[22] Delgado L.E., Escobar D.R., Hoyos D.M., Luna L., Pacheco-Lopez R., Ferro B. (2019). Nontuberculous mycobacteria in patients registered in a tuberculosis control program in Southwestern Colombia, 2014–2017. Interdiscip. J. Epidemiol. Public Health.

[23] Tichenor W.S., Thurlow J., McNulty S., Brown-Elliott B.A., Wallace R.J., Falkinham J.O. (2012). Nontuberculous Mycobacteria in household plumbing as possible cause of chronic rhinosinusitis. Emerg. Infect. Dis..

[24] Falkinham J.O. (2011). Nontuberculous mycobacteria from household plumbing of patients with nontuberculous mycobacteria disease. Emerg. Infect. Dis..

[25] Adékambi T., Drancourt M., Raoult D. (2009). The rpoB gene as a tool for clinical microbiologists. Trends Microbiol..

[26] CLSI (2018). Performance Standards for Susceptibility Testing of Mycobacteria, Nocardia spp., and Other Aerobic Actinomycetes.

[27] Ministerio de la protección social, Ministerio de Ambiente, Vivienda y Desarrollo Territorial (2007). Resolución Número 2115. República de Colombia. https://www.minambiente.gov.co/images/GestionIntegraldelRecursoHidrico/pdf/normativa/Res_2115_de_2007.pdf.

[28] Perez-Martinez I., Aguilar-Ayala D.A., Fernandez-Rendon E., Carrillo-Sanchez A.K., Helguera-Repetto A.C., Rivera-Gutierrez S., Estrada-Garcia T., Cerna-Cortes J.F., Gonzalez Y.M.J.A. (2013). Occurrence of potentially pathogenic nontuberculous mycobacteria in Mexican household potable water: A pilot study. BMC Res. Notes.

[29] Tortone C.A., Oriani D.S., Staskevich A.S., Oriani A.S., Gino L.M., Marfil M.J., Nava Vargas A., Gioffré A.K., Zumárraga M.J. (2019). Species diversity of non-tuberculous mycobacteria isolated from aquatic environments of General Pico city, Province of La Pampa (Argentina). Rev. Argent. Microbiol..

[30] Oriani A.S., Marfil M.J., Zumárraga M.J., Baldini M.D. (2019). Prevalence and species diversity of nontuberculous mycobacteria in drinking water supply system of Bahía Blanca City, Argentina. Int J. Mycobacteriol..

[31] Donohue M.J., Mistry J.H., Donohue J.M., O’Connell K., King D., Byran J., Covert T., Pfaller S. (2015). Increased Frequency of Nontuberculous Mycobacteria Detection at Potable Water Taps within the United States. Environ. Sci. Technol..

[32] Loret J.F., Dumoutier N. (2019). Non-tuberculous mycobacteria in drinking water systems: A review of prevalence data and control means. Int. J. Hyg. Environ. Health.

[33] Thomson R.M., Carter R., Tolson C., Coulter C., Huygens F., Hargreaves M. (2013). Factors associated with the isolation of Nontuberculous mycobacteria (NTM) from a large municipal water system in Brisbane, Australia. BMC Microbiol..

[34] Hussein Z., Landt O., Wirths B., Wellinghausen N. (2009). Detection of non-tuberculous mycobacteria in hospital water by culture and molecular methods. Int. J. Med. Microbiol..

[35] Le Dantec C., Duguet J.P., Montiel A., Dumoutier N., Dubrou S., Vincent V. (2002). Occurrence of mycobacteria in water treatment lines and in water distribution systems. Appl. Environ. Microbiol..

[36] Mi Z., Dai Y., Xie S., Chen C., Zhang X. (2015). Impact of disinfection on drinking water biofilm bacterial community. J. Environ. Sci..

[37] Norton C.D., LeChevallier M.W. (2000). A pilot study of bacteriological population changes through potable water treatment and distribution. Appl. Environ. Microbiol..

[38] Norton C.D., LeChevallier M.W., Falkinham J.O. (2004). Survival of Mycobacterium avium in a model distribution system. Water Res..

[39] LeChevallier M.W., Lowry C.D., Lee R.G., Gibbon D.L. (1993). Examining the Relationship Between Iron Corrosion and the Disinfection of Biofilm Bacteria. J. Am. Water Work. Assoc..

[40] Hoefsloot W., van Ingen J., Andrejak C., Angeby K., Bauriaud R., Bemer P., Beylis N., Boeree M.J., Cacho J., Chihota V. (2013). The geographic diversity of nontuberculous mycobacteria isolated from pulmonary samples: An NTM-NET collaborative study. Eur. Respir. J..

[41] Gupta N., Mittal A., Niyas V.K.M., Banerjee S., Ray Y., Kodan P., Malla S., Khot W., Fazal F., Singh B.K. (2020). Nontuberculous mycobacteria: A report of eighteen cases from a tertiary care center in India. Lung India.

[42] Gonzalez-Santiago T.M., Drage L.A. (2015). Nontuberculous Mycobacteria: Skin and Soft Tissue Infections. Dermatol. Clin..

[43] Sharma K., Gautam N., Sharma M., Dogra M., Bajgai P., Tigari B., Sharma A., Gupta V., Sharma S.P., Singh R. (2017). Ocular mycobacteriosis-dual infection of M. tuberculosis complex with M. fortuitum and M. bovis. J. Ophthalmic Inflamm. Infect..

[44] Kurokawa K., Harada N., Sasano H., Takagi H., Takei S., Nakamura A., Kamada K., Yoshida A., Kikuchi K., Takahashi K. (2020). Pulmonary infection due to fluoroquinolone-resistant Mycolicibacterium fortuitum: A case report. BMC Infect. Dis..

[45] Okamori S., Asakura T., Nishimura T., Tamizu E., Ishii M., Yoshida M., Fukano H., Hayashi Y., Fujita M., Hoshino Y. (2018). Natural history of Mycobacterium fortuitum pulmonary infection presenting with migratory infiltrates: A case report with microbiological analysis. BMC Infect. Dis..

[46] Pradier M., Boucher A., Robineau O., Chachaty E., Meybeck A., Senneville E. (2018). Mycobacterium mucogenicum bacteremia: Major role of clinical microbiologists. BMC Infect. Dis..

[47] Otchere I.D., Asante-Poku A., Osei-Wusu S., Aboagye S.Y., Yeboah-Manu D. (2017). Isolation and characterization of nontuberculous mycobacteria from patients with pulmonary tuberculosis in Ghana. Int. J. Mycobacteriol..

[48] Adékambi T., Foucault C., La Scola B., Drancourt M. (2006). Report of two fatal cases of Mycobacterium mucogenicum central nervous system infection in immunocompetent patients. J. Clin. Microbiol..

[49] Pereira S.G., Alarico S., Tiago I., Reis D., Nunes-Costa D., Cardoso O., Maranha A., Empadinhas N. (2019). Studies of antimicrobial resistance in rare mycobacteria from a nosocomial environment. BMC Microbiol..

[50] Ben Salah I., Adékambi T., Drancourt M. (2009). Mycobacterium phocaicum in therapy pool water. Int. J. Hyg. Environ. Health.

[51] Wethasinghe J., Hotu S., Taylor S., Anderson G., Wong C. (2015). Mycobacterium phocaicum and Mycobacterium avium-intracellulare in a patient with hot tub lung. Respirol. Case Rep..

[52] Cooksey R.C., Jhung M.A., Yakrus M.A., Butler W.R., Adékambi T., Morlock G.P., Williams M., Shams A.M., Jensen B.J., Morey R.E. (2008). Multiphasic approach reveals genetic diversity of environmental and patient isolates of Mycobacterium mucogenicum and Mycobacterium phocaicum associated with an outbreak of bacteremias at a Texas hospital. Appl. Environ. Microbiol..

[53] Zheng H.W., Pang Y., He G.X., Song Y.Y., Zhao Y.L. (2017). Antimicrobial Susceptibility Testing and Molecular Characterization of Mycobacterium fortuitum Isolates in China. Biomed. Environ. Sci..

[54] Kim S.Y., Moon S.M., Jhun B.W., Kwon O.J., Huh H.J., Lee N.Y., Lee S.H., Shin S.J., Kasperbauer S.H., Huitt G.A. (2019). Species Distribution and Macrolide Susceptibility of Mycobacterium fortuitum Complex Clinical Isolates. Antimicrob. Agents Chemother..

[55] Pérez-Vidal A. Estrategias de Implementación de los Planes de Seguridad del Agua para la Gestión del Riesgo en Sistemas de Abastecimiento de Agua Potable. Universidad del Valle 2013. https://opac.univalle.edu.co/cgi-olib/?infile=details.glu&loid=834909&rs=7139723&hitno=2.

[56] Parques Nacionales Naturales de Colombia Operación Farallones Acciones Interinstitucionales en Implementación para Controlar la Minería Ilegal de Oro 2015. https://www.parquesnacionales.gov.co/portal/es/operacion-farallones-acciones-interinstitucionales-en-implementacion-para-controlar-la-mineria-ilegal-de-oro-en-el-parque-nacional-natural-farallones-de-cali/.

[57] Pérez-Vidal A., Escobar-Rivera J.C., Torres-Lozada P. (2020). Development and implementation of a water-safety plan for drinking-water supply system of Cali, Colombia. Int. J. Hyg. Environ. Health.

[58] Montoya-Pachongo C., Douterelo I., Noakes C., Camargo-Valero M.A., Sleigh A., Escobar-Rivera J.C., Torres-Lozada P. (2018). Field assessment of bacterial communities and total trihalomethanes: Implications for drinking water networks. Sci. Total Environ..

